# Assessment of SARS-CoV-2-Infected Patients and Their Clinical Outcomes During the Third Wave in India: A Single-Center Observational Study

**DOI:** 10.7759/cureus.26807

**Published:** 2022-07-13

**Authors:** Praveen R Shahapur, Roopa Shahapur, Venkataramana Kandi, Tarun Kumar Suvvari, Sabitha Vadakedath

**Affiliations:** 1 Microbiology, BLDE (Deemed to be University) Shri B. M. Patil Medical College, Vijayapur, IND; 2 Dentistry, BLDE (Deemed to be University) Shri B. M. Patil Medical College, Vijayapura, IND; 3 Clinical Microbiology, Prathima Institute of Medical Sciences, Karimnagar, IND; 4 Medicine and Surgery, Rangaraya Medical College, Kakinada, IND; 5 Biochemistry, Prathima Institute of Medical Sciences, Karimnagar, IND

**Keywords:** vaccination, india, pandemic, third wave, clinical outcomes, covid-19, sars-cov-2

## Abstract

Background

The severe acute respiratory syndrome coronavirus-2 (SARS-CoV-2) that emerged from the Chinese mainland has spread throughout the world affecting the normal lives of the people. Both developed and developing nations have been equally affected and coronavirus disease-19 (COVID-19) resulted in the death of millions of people worldwide. The virus is undergoing mutations and is evolving into variants that are responsible for wave after wave. This study was carried out to assess the clinical outcomes of people infected with the novel virus during the third wave of the COVID-19 pandemic in India.

Methods

The study was carried out between November 2021 and January 2022 and included 100 consecutive patients attending the hospital attached to the BLDE (Deemed to be University) Shri B.M. Patil Medical College, Bijapur, Karnataka, South India. All patients included in the study returned a positive report in a real-time polymerase chain reaction (RT-PCR). The patient details collected included age, sex, cycle threshold (Ct) values for envelope (E)/nucleocapsid (N), and Orf1b (open reading frame 1b) genes, hospitalization status, vaccine status, C-reactive protein (CRP), D-dimer, interleukin-6 (IL-6), and final clinical outcome. The data were entered into Microsoft Office Excel sheets, and statistical inferences were drawn using SPSS 24 (IBM Corp., Armonk, NY).

Results

Of the 100 patients included in the study, only 14 (14%) patients were vaccinated. The patient's mean age was 34.22±17.50. Among the vaccinated patients, the majority had taken COVISHIELD™ (85.71%) compared to COVAXIN^®^ (14.29%). Only 14% of patients were symptomatic, and the mean Ct values among all the patients were 29.92±3.74 (E gene/N gene) and 27.6±4.78 (Orf1B gene). Eight (8%) patients were hospitalized, and all the patients recovered from the infection. Among the hospitalized patients, six (75%) were vaccinated. The mean age of the hospitalized patients was 43.8±14.25 years. The mean CRP, D-dimer, and IL-6 concentrations among the hospitalized patients were noted to be 22.375±16.58 mg/L, 654.325±577.24 ng/mL, and 5.075±2.15 ng/mL, respectively.

Conclusion

The study results demonstrate that despite unvaccinated status, most patients in the third wave had only suffered from asymptomatic infection. Moreover, people who developed a clinical infection and those who required hospitalization had an uneventful recovery irrespective of their vaccination status.

## Introduction

The coronavirus disease-19 (COVID-19) pandemic caused by the novel severe acute respiratory syndrome coronavirus-2 (SARS CoV-2) first emerged from the Chinese mainland in November 2019 and spread to all corners of the world [1,2]. After more than two years in existence, the SARS-CoV-2 has been regularly undergoing mutations and emerging into different variants [3]. Among the variants that have emerged, the Delta variant was considered the most virulent. However, the recently identified omicron variant had replaced all the previous variants including the delta.

The omicron was first reported by South African scientists and the initial observations were that the virus is extremely transmissible and causes less severe infections than the delta infections [4]. Surprisingly, the Omicron variant developed into several sublineages, and more recently, recombinants formed from delta and omicron and omicron sublineages have been continuously emerging [5]. The omicron variant and its sublineages have been noted to be increasingly transmissible than the previous variants and have the potential to evade immune responses and be resistant to monoclonal antibody therapy [6,7].

The whole world is still trying to cope with the omicron variant's resurgence despite the availability of a vaccine. In India, although the third wave was milder than the second wave caused by the delta variant, the cause of concern was the frequency of breakthrough infections [8,9]. Most infections during the third wave have been caused by the omicron variant that is more than capable of evading immune responses owing to multiple mutations at the spike protein and receptor binding domain regions of the viral genome [10,11].

However, due to the availability of vaccines, people were presumed to have protective antibodies. Despite that, the third wave saw an exponential increase in the number of infections and numerous breakthrough infections. This could be attributed to the incomplete vaccinations wherein people who were vaccinated had received only a single dose instead of two doses [12]. Also, there is vaccine hesitancy among people which contributed to low vaccination rates, especially in the initial days of vaccination [13,14]. The present study was undertaken to assess the clinical outcomes among people infected with SARS-CoV-2 during the third wave in India.

## Materials and methods

This was an observational study that was carried out between November 2021 and January 2022. A convenience and consecutive sampling method were applied wherein 100 patients who attended the tertiary care hospital attached to the BLDE (Deemed to be University) Shri B. M. Patil Medical College, Hospital and Research Center, Bijapur, Karnataka, South India were included in the study. The study was approved by the institutional ethics committee of Shri B.M. Patil Medical College, Hospital and Research Center (IEC/591-A/21-12-2021).

All patients who were positive for SARS-CoV-2 by real-time polymerase chain reaction (RT-PCR) were included in the study. The patient details collected included age, sex, cycle threshold (Ct) values for envelope (E)/nucleocapsid (N), and Orf1b (open reading frame 1b) genes, hospitalization status, vaccine status, serum activities of C-reactive protein (CRP), D-dimer, interleukin-6 (IL-6), and final clinical outcome.

The serum activities of CRP were estimated using Beckman Coulter Access 2 (Beckman Coulter, Inc., USA), an automated chemistry analyzer by the immunoturbidometric method. Serum IL-6 levels were measured by chemiluminescence immunoassay (Beckman Coulter Access 2). The serum D-dimer activities were measured by mini VIDAS® (bioMérieux, SA) using an enzyme-linked fluorescent assay principle. Clinical data were obtained/collected from medical records on admission.

The data were entered into Microsoft Office 2019 Excel sheet (Microsoft® Corp., Redmond, WA), and statistical inferences were drawn using SPSS software version 24 (IBM Corp., Armonk, NY). The quantitative data were represented as percentage, mean, and standard deviation (SD). The t-test was used to analyze quantitative data and the significance threshold of the p-value was set at <0.05.

## Results

Of the 100 patients included in the study, only 14 (14%) patients were vaccinated. The patient's mean age was 34.22±17.50. Male patients (72%) accounted for more infections in comparison to female patients (28%). Among the vaccinated patients, a majority had taken COVISHIELD™ (85.71%) compared to those who received COVAXIN® (14.29%). Only 14% of patients were symptomatic, and the mean Ct values among all the patients were 29.92±3.74 (E gene/N gene) and 27.6±4.78 (Orf1B gene). Only eight (8% ) patients were hospitalized, and all the patients recovered from the infection. There was only a slight difference in the Ct values among the unvaccinated (29.74±3.96 [E gene/N gene] and 27.30±4.84 [Orf1B gene]) and vaccinated people (31±1.56 [E gene/N gene] and 29.42±3.83 [Orf1B gene]) (p-value 0.244).

Among the hospitalized patients, 6 (75%) were vaccinated. The mean age of the hospitalized patients was 43.8±14.25 years. The Ct values among the hospitalized patients were 26±6 (E gene/N gene) and 26.75±6.73 (Orf1B gene). The mean CRP, D-dimer, and IL-6 concentrations among the hospitalized patients were 22.375±16.58 mg/L, 654.325±577.24 ng/mL, and 5.075±2.15 ng/mL, respectively. The demographic and clinical details of the patients are delineated in Table 1.

**Table 1:** The symptomatology, vaccination status, clinical profile, and outcomes among the study participants COVAXIN®: Covaxin vaccine; COVISHIELD™: Covishield vaccine; CRP: C-reactive protein; IL-6: Interleukin-6

Parameter	Variable	Number of Individuals (n=100) (%)
Sex	Male	72 (72%)
	Female	28 (28%)
Vaccination status	Vaccinated	14 (14%)
	Unvaccinated	86 (86%)
Type of vaccine	COVAXIN^®^	2 (14.29%)
	COVISHIELD™	12 (85.71%)
Symptomatic status	Symptomatic	14 (14%)
	Asymptomatic	86 (86%)
Hospitalization	Yes	8 (8%)
	No	92 (92%%)
Mortality	Vaccinated	0 (00%)
	Unvaccinated	0 (00%)
	Hospitalized	0 (00%)
Clinical parameters among hospitalized patients	CRP (mg/L)	22.375±16.58
	D-dimer (ng/mL)	654.325±577.24
	IL-6 (ng/mL)	5.075±2.15

The Ct values were found statistically significant among unvaccinated versus hospitalized patients (p=0.016), and all patients versus those who were hospitalized (p= 0.001). The Ct values of the PCR test of all the study participants are detailed in Table 2.

**Table 2:** The comparison of Ct values among the vaccinated and unvaccinated patients. Ct: Cycle threshold; E gene: Gene coding for envelope; N gene: Gene coding for nucleocapsid; Orf1b: open reading frames 1b*: Statistically significant

Status of vaccination (n)	Ct values (Mean±SD)		P-value
	E Gene/N gene	Orf1B gene	
Vaccinated (14)	31±1.56	29.42±3.83	0.244 (vaccinated Vs unvaccinated)
Un vaccinated (86)	29.74±3.96	27.30±4.84	0.016* (unvaccinated Vs hospitalized)
Hospitalized (8)	26±6	26.75±6.73	0.001* (hospitalized Vs all patients)
All participants (100)	29.92±3.74	27.6±4.78	0.285 (vaccinated Vs all patients)

## Discussion

The novel SARS-CoV-2, the causative agent for COVID-19, has resulted in severe morbidity and mortality throughout the world. With the emergence of newer viral variants, it was tough for the governments to control the spread of infections. Moreover, the newer viral variants were noted to possess increased transmissibility as compared to the preceding ones. Despite the availability of a vaccine, and extensive vaccination drives among people, there were several reports of breakthrough infections. Studies have confirmed that the omicron variant had more mutations in comparison to the previous ones, which contributed to its immune escape and vaccine resistance. In India, the second wave of the pandemic was extremely devastating that claimed thousands of people’s lives. This wave was attributed to the delta variant of the SARS-CoV-2, which was considered the most virulent variant that was responsible for severe infections, especially among debilitated patients [15,16].

Given the availability of the mRNA-based vaccines, and with increased vigilance, social distancing, and awareness, the infections were brought under control [17,18]. However, the emergence and spread of the Omicron variant had brought in the third wave in India and elsewhere in the world [19]. Due to the presence of unique mutations, this virus variant was noted to be more transmissible, and treatment-resistant as compared to the delta variant. Moreover, within a few months after its discovery, the omicron variant not only spread throughout the world but also replaced the delta variant [20].

The omicron variant was found to evade natural immunity as well as vaccine-induced immunity and was being increasingly associated with breakthrough infections. The present study was carried out to assess the impact of the third wave, which was potentially caused by the omicron variant. The results have indicated that most of the people who acquired infections only suffered from mild infections. There was no death reported within the study group.

The study results have demonstrated that there was a significant association of Ct values in the unvaccinated group with hospitalization (p=0.016). Also, it was noted that the people who were hospitalized had significantly lower Ct values as compared to all the patients in the study (p<0.001). This suggests that the vaccinated people could suffer from infections with lower viral loads as compared to the unvaccinated population. Despite immune evasion, lower vaccination rates, and waning of vaccine-induced protection, the third wave was less severe in comparison to the second wave which was caused by the delta variant.

A similar observation was made by a South African study, wherein the omicron variant was discovered for the first time [21]. This study noted that the disease severity, hospitalizations, and deaths were significantly lower as compared to the preceding waves caused by the delta and other variants. The current study and a few previous ones that assessed the omicron infections have confirmed the fact that although the omicron variant resulted in increased transmissibility and raised infection rates and was noted to be immune evasive and antibody resistant, it resulted in milder infections and fewer hospitalizations [22].

A multicentric analysis of clinical outcomes among the omicron infected patients revealed that the vaccinated persons and people who received booster doses are protected against hospitalization and death (OR 0.30, CI 0.15-0.57, p=0.0003) [23]. A prospective cohort study from the United Kingdom that observed clinical outcomes among the long-term care facility people has noted reduced severity of infections (hospitalizations (0·64, 95% CI 0·41-1·00; p=0·051) and mortality (aHR 0·68, 0·44-1·04; p=0·076) caused by the omicron as compared to the previous variants [24]. Interestingly, observations from a recent omicron-related outbreak in the Chinese-administered Hong Kong region showed that the unvaccinated (10,076 per million population) and people aged over 60 suffered increased mortality as compared to people who received booster doses of vaccination (473 per million population) [25].

Limitations

Although the study participants were recruited during the third wave of the COVID-19 pandemic that occurred in India, the confirmation of the viral variant was not completed by next-generation sequencing (NGS). Moreover, the numbers of study participants were not equally selected based on their vaccination status. More than 85% of the study participants were unvaccinated. Therefore, the protection conferred by the vaccination was not completely evident from the study results. Also, the study participant's previous history concerning the SARS-CoV-2 infection, co-morbidities, and other underlying health conditions were not recorded and the clinical outcomes of the previous and present waves were not compared.

## Conclusions

The study results point to the fact that the third wave of the COVID-19 pandemic resulted in less morbidity and mortality. Despite the vaccination being available, most participants in the study group were unvaccinated. Moreover, irrespective of their vaccination status, most patients developed an asymptomatic infection. The results noted zero deaths among the infected patients and those who were hospitalized also recovered uneventfully. The unvaccinated group had lower Ct values suggesting a higher viral load that could potentially predispose them to hospitalization. It may be inferred from the results of the current study that most people could have been exposed to the virus because of community transmission. Therefore, both vaccinated and unvaccinated people suffered from clinically mild infections and had an uneventful recovery.
